# Increasing stability of water-soluble PQQ glucose dehydrogenase by increasing hydrophobic interaction at dimeric interface

**DOI:** 10.1186/1471-2091-6-1

**Published:** 2005-02-16

**Authors:** Shunsuke Tanaka, Satoshi Igarashi, Stefano Ferri, Koji Sode

**Affiliations:** 1 Department of Biotechnology, Tokyo University of Agriculture and Technology, 2-24-13 Naka-machi, Koganei, Tokyo, 184-8588, Japan

## Abstract

**Background:**

Water-soluble quinoprotein glucose dehydrogenase (PQQGDH-B) from *Acinetobacter calcoaceticus *has a great potential for application as a glucose sensor constituent. Because this enzyme shows no activity in its monomeric form, correct quaternary structure is essential for the formation of active enzyme. We have previously reported on the increasing of the stability of PQQGDH-B by preventing the subunit dissociation. Previous studies were based on decreasing the entropy of quaternary structure dissociation but not on increasing the interaction between the two subunits. We therefore attempted to introduce a hydrophobic interaction in the dimeric interface to increase the stability of PQQGDH-B.

**Results:**

Amino acid residues Asn340 and Tyr418 face each other at the dimer interface of PQQGDH-B, however no interaction exists between their side chains. We simultaneously substituted Asn340 to Phe and Tyr418 to Phe or Ile, to create the two mutants Asn340Phe/Tyr418Phe and Asn340Phe/Tyr418Ile. Furthermore, residues Leu280, Val282 and Val342 form a hydrophobic region that faces, on the other subunit, residues Thr416 and Thr417, again without any specific interaction. We simultaneously substituted Thr416 and Thr417 to Val, to create the mutant Thr416Val/Thr417Val. The temperatures resulting in lose of half of the initial activity of the constructed mutants were increased by 3–4°C higher over wild type. All mutants showed 2-fold higher thermal stability at 55°C than the wild-type enzyme, without decreasing their catalytic activities. From the 3D models of all the mutant enzymes, the predicted binding energies were found to be significantly greater that in the wild-type enzyme, consistent with the increases in thermal stabilities.

**Conclusions:**

We have achieved via site-directed mutagenesis the improvement of the thermal stability of PQQGDH-B by increasing the dimer interface interaction. Through rational design based on the quaternary structure of the enzyme, we selected residues located at the dimer interface that do not contribute to the intersubunit interaction. By substituting these residues to hydrophobic ones, the thermal stability of PQQGDH-B was increased without decreasing its catalytic activity.

## Background

Water-soluble quinoprotein glucose dehydrogenase (PQQGDH-B) from *Acinetobacter calcoaceticus *has great potential for application as a constituent of an electron mediator-type glucose sensor. The conventionally utilized enzyme for glucose measurement, glucose oxidase (GOD), inherently utilizes oxygen as its electron acceptor during the oxidation of glucose. In contrast, PQQGDH-B is completely independent of oxygen, resulting in improved accuracy and rapidity during glucose measurement. However, because the substrate specificity and stability of PQQGDH-B remain somewhat inferior to those of GOD, we have been engaged in the improvement of these enzymatic properties through protein engineering to further increase the application of PQQGDH-B in glucose monitoring systems [1-17].

The subunit structure of PQQGDH-B was determined to be homodimeric with no activity observed in its monomeric form [17]. Correct quaternary structure is essential for the formation of active enzyme and dissociation of the dimer conformation triggers inactivation of the enzyme. We have previously reported on the increasing of the stability of PQQGDH-B against the dissociation of quaternary structure by chemical cross-linking [12], by constructing tethered enzyme [13], and by introducing Cys residues at the dimer interface to form a novel intersubunit disulfide bond [14]. All of these attempts were based on decreasing the entropy by decreasing the possibility of dimer dissociation, but no attempts had been made to increase the interaction between the two subunits.

In this paper, we report on our rational designing of hydrophobic interaction in the dimer interface to increase the stability of PQQGDH-B. We identified protein regions at the dimer interface where potential novel hydrophobic interactions could be introduced by amino acid substitution, thereby increasing the stability.

## Results

### Modeling novel dimer hydrophobic core

**Figure 1:**
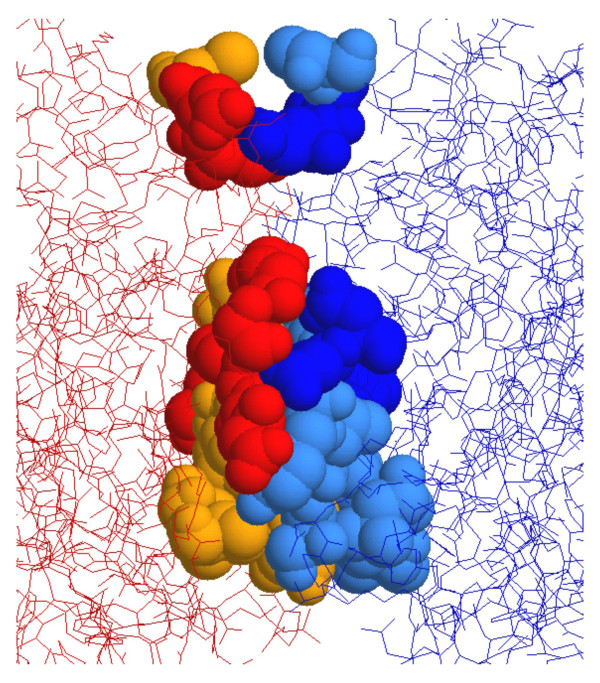
**The amino acid residues located at the dimer interface. **The two subunits are represented in red and blue, respectively, using the RasMol molecular visualization software 26. The 19 amino acid residues at the interface are shown in space filling format, of which 8 residues (orange and light blue) are predicted not be involve in hydrogen bond formation, electrostatic interaction, or hydrophobic interaction at the interface.

**Figure 2:**
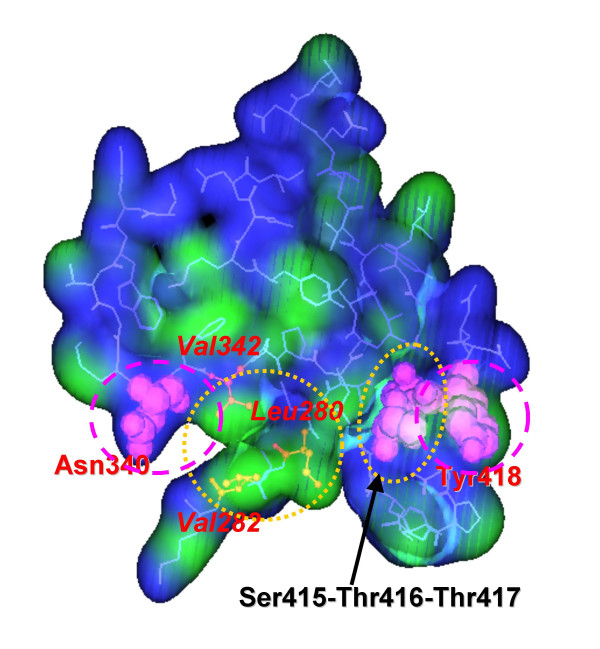
**Hydrophobicity of PQQGDH-B dimer interface. **Hydrophobic regions are shown in green and hydrophilic regions are shown in blue. Residues that have been substituted in this study are indicated. The structural images were generated using Molecular Operating Environment.

Among 19 amino acid residues located at the dimer interface, 8 residues were predicted not to be involved in the formation of hydrogen bonds, electrostatic interactions, or hydrophobic interactions (Fig. 1). We focused on the residues Asn340, Tyr418, Thr416, and Thr417, as they are not involved in the formation of the active site cavity and are therefore suitable candidates for amino acid substitution. Although residues Asn340 and Tyr418 face each other on the surface of the dimer interface (Fig. 2), no interaction exists between their side chains. We therefore simultaneously substituted Asn340 to Phe and Tyr418 to Phe or Ile to create the mutants Asn340Phe/Tyr418Phe and Asn340Phe/Tyr418Ile, respectively. Furthermore, the hydrophobic region composed of residues Leu280, Val282, and Val342 faces residues Thr416 and Thr417, again with no specific interaction. We therefore substituted Thr416 to Val and Thr417 to Val to create the double mutant Thr416Val/Thr417Val.

### Characterization of mutant enzymes

**Figure 3:**
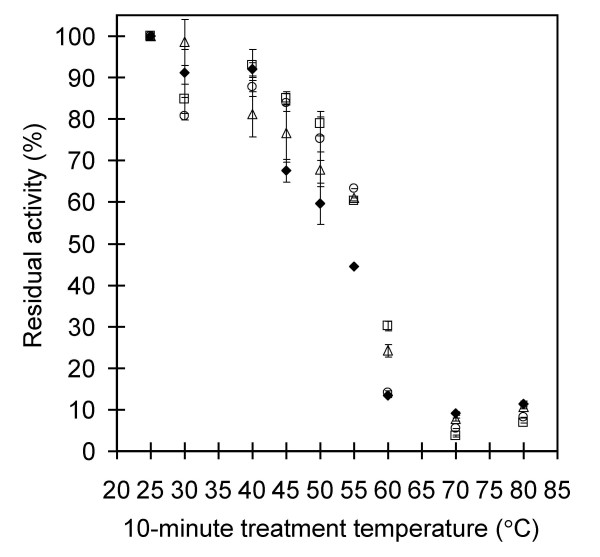
**Thermal stability of wild-type and mutant PQQGDH-Bs. **Residual activity was measured at 25°C after 10-min incubations at different temperatures of the following protein samples (0.075 μg/mL): Wild-type ◆, Asn340Phe/Tyr418Phe □, Asn340Phe/Tyr418Ile △, and Thr416Val/Thr417Val ○.

**Figure 4:**
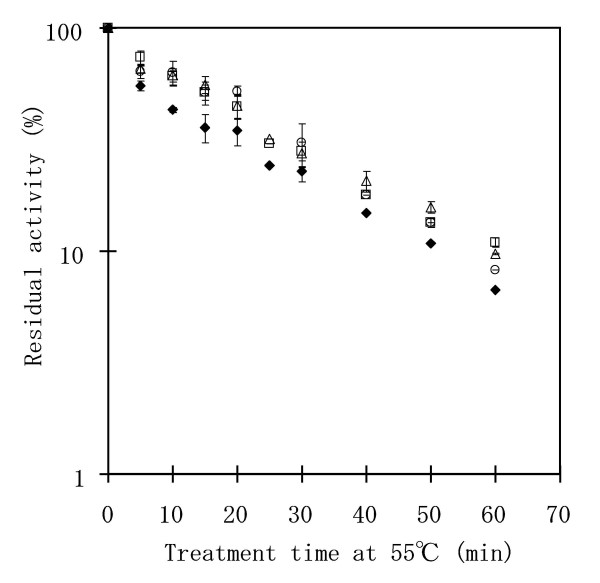
**Time course of thermal inactivation of wild-type and mutant PQQGDH-Bs. **Protein samples (0.075 μg/mL) were incubated at 55°C and aliquots were taken at different times to measure residual activity. The analyzed samples contained the following PQQGDH-Bs: Wild-type ◆, Asn340Phe/Tyr418Phe □, Asn340Phe/Tyr418Ile △, Thr416Val/Thr417Val ○.

The activity and stability of each of the three constructed double mutant enzymes were then analyzed. All three mutant enzymes showed slightly higher thermal stability than wild-type PQQGDH-B upon incubation for 10 min at various temperatures (Fig. 3). The temperatures resulting in lose of half of the initial activity were shifted by approximately 3°C higher in the mutants compared to the wild type (Table 1). As the time course of thermal inactivation at 55°C follows first-order kinetics (Fig. 4), half-lives were calculated using logarithmic regression of residual activity. The wild-type enzyme inactivates at 55°C with a half-life of 9.5 ± 1.4 min, while all three double mutants showed greater thermal stability, with half-lives of 5–6°C greater (Table 1).

**Table 1:** Kinetic parameters and thermal stability of PQQGDH-Bs.

	*V*max(U/mg)	*K*m(mM)	T_h_(°C)^a^	t_1/2 _(min)^b^
Wild type	3030	20	53.9 ± 0.9	9.5 ± 1.4
Asn340Phe/Tyr418Phe	3100	20	57.7 ± 0.4	14.9 ± 1.1
Asn340Phe/Tyr418Ile	2500	20	57.5 ± 0.3	15.5 ± 1.5
Thr416Val/Thr417Val	2800	16	56.5 ± 0.9	14.8 ± 1.4

^a ^T_h _represents the temperature at which half the initial activity is lost in 10 min.

^b ^t_1/2 _represents the half-life at 55°C.

Investigation of the kinetic parameters of the wild-type and mutant enzymes shows that the mutations did not significantly affect the overall kinetic properties of PQQGDH-B (Table 1). The specific activities of Asn340Phe/Tyr418Phe (3100 U/mg), Asn340Phe/Tyr418Ile (2500 U/mg), and Thr416Val/Thr417Val (2800 U/mg) were close to that of the wild-type enzyme (3030 U/mg). Except for Thr416Val/Thr417Val, which had a Km value of 16 mM, the mutants had Km values identical to 20 mM Km value of the wild-type enzyme.

## Discussion

PQQGDH-B is a 6-blade β-propeller protein, with each blade consisting of a 4-stranded anti-parallel β-sheet (W-motif) [18]. The strands in each W-motif are labeled A-D from the inside to the outside of the molecule [18,19]. All strands are connected by loops, which are named according to the strands they connect. Based on PQQGDH-B structural information, these loop regions have been associated with a number of important functions, such as substrate binding, co-factor binding, and formation of the enzyme active site. As with other β-propeller proteins, the catalytic site and substrate-binding pocket of PQQGDH-B is made up of the cleft formed by loops DA and BC [20]. The enzyme surface composed of loops AB and CD is therefore located opposite the functional region. In the present study, we have introduced mutations at Asn340, Tyr418, Thr416, and Thr417, which are all located in the loop 5CD region. Considering that these residues are located far from the functional region and do not contribute in the structure of functional region, it is not surprising that their substitutions did not significantly alter the enzyme's kinetic parameters, particularly its catalytic activity.

**Figure 5:**
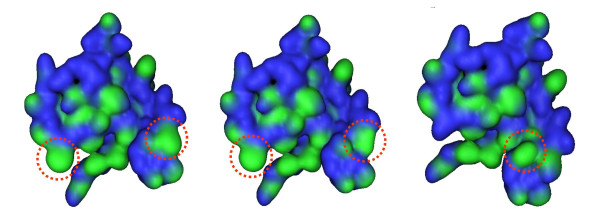
**Hydrophobicity of mutant PQQGDH-B dimer interface. **Hydrophobic regions are shown in green while hydrophilic regions are shown in blue. The interfaces shown are those of the Asn340Phe/Tyr418Phe (left), Asn340Phe/Tyr418Ile (middle), and Thr416Val/Thr417Val (right) mutants of PQQGDH-B, with their respective mutation sites circled. The structural images were generated using Molecular Operating Environment.

Based on the predicted structure of the mutant enzymes shown in Figure 5, the binding energy of each subunit was calculated, using both the AMBER89 and CHARMM22 force fields, and compared with those of the wild-type enzyme (Table 2). As expected, the increases in hydrophobicity at the subunit interface resulted in increases in their binding energies calculated by both methods. Estimation of the number of hydrophobic interactions based on these same predicted models revealed 4 to 5 novel hydrophobic interactions at the interface of all the mutants, while none were found in the wild-type one. These results based on structural predictions are consistent with the observed improvements in thermal stability.

**Table 2:** Predicted binding energy of each mutant subunit calculated using AMBER89 and CHARMM22 force fields.

	Binding energy (kcal/mol)	
	
	AMBER89	CHARMM22
Wild type	-249	-118
Asn340Phe/Tyr418Phe	-291	-134
Asn340Phe/Tyr418Ile	-273	-126
Thr416Val/Thr417Val	-278	-131

The addition of hydrophobic interactions in other enzymes has been reported to result in thermostability increases of 2 to 10°C [21-24], comparable to the results of the current study. Recent observations of enzymes from thermophilic organisms indicate that their extraordinary thermal stability is due to hydrophobic interactions. Oligomeric enzyme stability was also reported to be improved through engineering to increase the hydrophobic interaction at the oligomer interface [25]. However, these studies were based on homology analyses between mesophilic and thermophilic bacteria together with random mutagenesis library analyses [21-24]. Although some sequences have been found to be homologous to PQQGDH-B, these are all putative ORFs with no functional information reported. Therefore, there exists no reliable template to help improve the stability of this enzyme by increasing the dimer interface interaction.

## Conclusions

We improved PQQGDH-B's thermal stability by increasing the dimer interface interaction through rational design based on its quaternary structure. We demonstrated that this can be achieved by selecting residues located at the dimer interface that do not contribute to the intersubunit interaction and substituting them to hydrophobic ones via site-directed mutagenesis. In each case tested, the enzyme's thermal stability was increased without decreasing its catalytic activity. This rational design approach will provide relevant information for future designs by combining with other mutant PQQGDH-Bs with narrowed substrate specificity and improved catalytic efficiency.

## Methods

### Chemicals

Glucose, phenazine methosulfate (PMS), 2,6-dichlorophenolindophenol (DCIP), and magnesium chloride were obtained from Kanto Kagaku (Tokyo, Japan), 3-(N-morpholino) propane sulfonate (MOPS) from Dojin (Kumamoto, Japan), and pyrroloquinoline quinone from Mitsubishi Gas Chemical Company (Tokyo, Japan). All other regents were of analytical grade. *Kpn*I was obtained from TOYOBO (Osaka, Japan) and *Hin*dIII from New England BioLabs (Beverly, USA).

### Site-directed mutagenesis

The structural gene for wild-type PQQGDH-B was previously amplified by polymerase chain reaction (PCR) and inserted into the expression vector pTrc99A (Pharmacia) to create pGB [15]. A 1.2-kbp *Kpn*I-*Hin*dIII fragment containing the PQQGDH-B gene was transferred from pGB to pKF18k and mutagenesis was carried out with the Mutan-Express Km kit (Takara) according to the manufacturer's instructions with the oligonucleotides Asn340Phe (5'-GGTGGGACAAA**GAA**TTTACCAGTCC-3'), Tyr418Phe (5'-CGGTACAGCGTCATC**AAA**AGTAGTGC-3'), Tyr418Ile (5'-CGGTACAGCGTCATC**AAT**AGTAGTGC-3'), and Thr416Val/Thr417Val (5'-CAGCGTCATCATA**AACAAC**GCTATAAGTTGGATC-3'). The mutations (underlined) were confirmed by automated DNA sequencing (ABI PRISM Genetic analyzer 310, Applied Biosystems). The mutated genes were digested with *Kpn*I and *Hin*dIII and were replaced into pGB to construct expression vectors containing mutated PQQGDH-B. Numbering of the amino acid positions starts from the first residue of the signal peptide (24 residues).

### Enzyme preparation and assay

The PQQGDH-B genes were expressed in *Escherichia coli *and the enzymes purified as previously reported [15,16]. Following a 30-min preincubation in 10 mM MOPS-NaOH (pH 7.0) containing 1 μM PQQ and 1 mM CaCl_2 _at room temperature (25°C) to produce the holoenzyme, GDH activity was measured by using 0.6 mM PMS and 0.06 mM DCIP. The enzyme activity was determined by measuring the decrease in absorbance of DCIP at 600 nm.

### Analysis of PQQGDH-B stability

The thermal stability of wild-type and mutant PQQGDH-B was determined with 0.075 μg/mL protein, as previously reported [15]. Thermal inactivation experiments were carried out by incubating each holoenzyme in 200 μL of 10 mM MOPS-NaOH, pH 7.0, at 55°C. Aliquots were taken every 5 min and kept at 4°C for 2 min, followed by incubation at room temperature for 30 min. The residual enzyme activity was determined as described above. Since the initial time course for thermal inactivation at 55°C followed first-order kinetics, the thermal stability of each mutant enzyme was expressed as a half-life. The thermal stability of Asn340Phe/Tyr418Phe, Asn340Phe/Tyr418Ile and Thr416Val/Thr417Val were also determined by incubating purified enzyme at various temperatures for 10 min. The residual activities were determined as described above, and were compared with the initial activities.

### Prediction of three-dimensional structure and quaternary-dimensional structures

Three-dimensional and quaternary structures were predicted using Molecular Operating Environment (MOE) (Chemical Computing Group Inc., Quebec, Canada). By using the available PDB data of the wild-type PQQGDH-B, 1QBI [18], we made the appropriate substitutions with all possible side-chain orientations to predict the structures of the Asn340Phe/Tyr418Phe, Asn340Phe/Tyr418Ile and Thr416Val/Thr417Val mutants. After addition of hydrogen atoms to the PQQGDH-B structure and optimization of orientation of some hydrogen atoms by MOE, the structures were subjected to energy minimization using the AMBER89 or CHARMM22 force field within the MOE program until the final energy gradient was < 0.01 kcal/mol·Å.

## Authors' contributions

ST carried out the site-directed mutagenesis of PQQGDH-B as well as the preparation and characterization of the resulting proteins. SI carried out the 3D modeling and participated in the design of the study. SF participated in interpretation of the results and in drafting the manuscript. KS conceived of the study, participated in its design and coordination, as well as in drafting the manuscript. All authors read and approved the final manuscript.
